# Oral nicotine aggravates endothelial dysfunction and vascular inflammation in diet-induced obese rats: Role of macrophage TNFα

**DOI:** 10.1371/journal.pone.0188439

**Published:** 2017-12-13

**Authors:** Chang Liu, Ming-Sheng Zhou, Yao Li, Aimei Wang, Kiranmai Chadipiralla, Runxia Tian, Leopoldo Raij

**Affiliations:** 1 Department of Endocrinology, First Affiliated Hospital, Jinzhou Medical University, Jinzhou, P.R. of China; 2 Department of Physiology, Shenyang Medical University, Shenyang, P.R. of China; 3 Department of Physiology, Jinzhou Medical University, Jinzhou, P.R. of China; 4 Nephrology-Hypertension Section, University of Miami Miller School of Medicine, Miami VAMC, Miami, Florida, United States of America; University of Southampton, UNITED KINGDOM

## Abstract

Obesity and cigarette smoke are major cardiovascular (CV) risk factors and, when coexisting in the same individuals, have additive/synergistic effects upon CVD. We studied the mechanisms involved in nicotine enhancement of CVD in Sprague Dawley rats with diet–induced obesity. The rats were fed either a high fat (HFD) or normal rat chow diet with or without nicotine (100 mg/L in drinking water) for 20 weeks. HFD rats developed central obesity, increased systolic blood pressure (SBP), aortic superoxide (O_2_^-^) production, and impaired endothelial nitric oxide synthase (eNOS) and endothelium-dependent relaxation to acetylcholine (EDR). Nicotine further increased SBP, O_2_^-^ and impaired eNOS and EDR in obese rats. In the peritoneal macrophages from obese rats, tumor necrosis factor (TNF) α, interleukin 1β and CD36 were increased, and were further increased in nicotine-treated obese rats. Using PCR array we found that 3 of 84 target proinflammatory genes were increased by 2–4 fold in the aorta of obese rats, 11 of the target genes were further increased in nicotine-treated obese rats. HUVECs, incubated with conditioned medium from the peritoneal macrophages of nicotine treated-obese rats, exhibited reduced eNOS and increased NADPH oxidase subunits gp91phox and p22phox expression. Those effects were partially prevented by adding anti-TNFα antibody to the conditioned medium. Our results suggest that nicotine aggravates the CV effects of diet–induced obesity including the oxidative stress, vascular inflammation and endothelial dysfunction. The underlying mechanisms may involve in targeting endothelium by enhancement of macrophage-derived TNFα.

## Introduction

Cigarette smoke is the most common cause of preventable morbidity and mortality worldwide, and an independent risk factor for cardiovascular (CV) diseases and type 2 diabetic mellitus[1, 2]. We and others have demonstrated the importance of chemically stable compounds, present in the gas phase of cigarette smoke, in mediating endothelial injury and atherosclerosis[3–5]. Nicotine, one of the major active compounds of cigarette smoke, has been shown to have adverse effects upon the CV system[5, 6], including autonomic imbalance, endothelial dysfunction and impaired coronary blood flow. It has been documented that nicotine, at concentration similar to that found in smokers’ blood, modifies lipid metabolism and impairs endothelial function in animals[7].

Vascular endothelium plays an important role in the maintenance of CV health. Endothelial dysfunction is a key feature of early atherosclerotic lesions and predictive of CV prognosis in both human and animal models[8, 9]. Endothelial cells are major targets of inflammatory cytokines released from various immune cells and vascular cells[10]. It has been shown that inflammatory cytokines, such as tumor necrosis factor (TNF)*α*, interact with endothelial cells or vascular smooth muscle cells to induce endothelial nitric oxide dysfunction, reactive oxygen species (ROS) production and vascular smooth muscle cell proliferation, resulting in endothelial dysfunction and promotion of CV diseases (CVD)[11–13].

Active macrophages are the main source of inflammatory cytokines. Macrophages *via* their scavenger receptors take up oxidized LDL (oxLDL) and other lipids, undergo activation, and produce various cytokines[14]. The macrophages also produce an oxidative state that promotes the oxidation of LDL, activation of endothelial cells and monocyte migration into the vascular wall, initiation of vascular inflammation and progression of atherosclerosis[15, 16]. Recently, we[3] have shown that nicotine can synergize with oxLDL to increase macrophage expression of scavenger receptor CD36. Nicotine in the presence of oxLDL promoted macrophage activation and production/release of multiple pro-inflammatory cytokines in vitro including TNFα, interleukin 6 (IL6) and monocyte chemoattractant protein (MCP)1 and accelerated atherosclerosis in vivo through CD36-dependent mechanisms[3].

Obesity is a chronic low-grade inflammatory disease associated with increased oxidative stress and plasma levels of various atherogenic lipids including oxLDLs[17, 18]. Epidemiological studies indicate that the combination of obesity and smoking results in significant increase in total death and CV death risk in both men and women[19, 20]. Here, we hypothesize that nicotine augments the CV effects of diet-induced obese rats via promoting macrophages to produce/release inflammatory cytokines such as TNFα, resulting in endothelial dysfunction via disrupting the balance between eNOS/NO and ROS in the vasculature.

## Materials and methods

### Animals and experimental protocols

The animals were housed in facilities accredited by the American Association for Accreditation of Laboratory Animal Care and by the Chinese Association for Accreditation of Laboratory Animal Care. The Institutional Animal Care and Use Committee at the Miami VA Medical Center and Jinzhou Medical University approved the studies. All procedures were performed in accordance with the Guide for the Care and Use of Laboratory Animals published by the US National Institutes of Health (Eighth Edition, the Guide, NRC 2011). Six-week-old Sprague-Dawley male rats were purchased from Harlan Sprague-Dawley Inco. (Indianapolis, IN) and maintained under controlled conditions of light, temperature, and humidity. After having 2 weeks to accommodate to the new environment, the rats were randomly divided into 4 groups and treated for 20 weeks (n = 6–7): NFD (normal fat diet): fed a normal rat chow diet (17% caloric from fat); Nic: fed a NFD diet with nicotine (100 mg/L in drinking water); HFD (high fat diet): fed a high fat diet (47% caloric from fat); HFD/Nic: fed a HFD plus nicotine treatment. Body weight was measured every week. Systolic blood pressure (SBP) was measured in the conscious rats by the tail-cuff method. At the end of the study, the rats were starved overnight, fasted plasma glucose was measured by blood glucose meter. Fasted plasma cholesterol and nonesterified free fatty acids (NFFA) were determined by cholesterol assay kit (Wako Diagnostics, Richmond, VA) and NFFA assay kit, respectively (Wako Diagnostics, Richmond, VA). The rats were anesthetized by sodium pentobarbital (50 mg/kg IP) and euthanized by decapitation; the heart, aortas and total abdominal fat were weighed. The heart weight/body weight and aortic weight/cm (arch of thoracic aorta to the origin of mesenteric artery) were used as indices of cardiac and aortic hypertrophy.

### Histological analysis

The aorta (1 cm below the highest point of the aortic arch) was isolated. The specimens from the thoracic aorta were fixed in 4% paraformaldehyde in phosphate-buffered saline. The 4-μm thick sections were cut and stained with hematoxylin and eosin. Four nonconsecutive digital images per animal were acquired with a LC Evolution camera (Media Cybernetics, Inc., Bethesda, Maryland, USA) and Olympus BX51 microscope (Leeds Precision Instruments, Inc., Minneapolis, Minnesota, USA) and analyzed with Image-Pro Plus version 6.0 software (Media Cybernetics, Inc.). The radial thickness of the media was measured.

### Isolation of peritoneal macrophages

Another set group of rats as described above were used to isolate the macrophages from the peritoneal cavity, the rats were injected intraperitoneally (I.P) with 5 ml of 3% thyoglycolate medium to induce the macrophages to enter the peritoneal cavity. On day 5 post-injection, the rats were anesthetized with sodium pentobarbital (50 mg/kg I.P) and injected with 5 to 10 ml cold PBS with 10 mmol/L EDTA to rinse the macrophages from the peritoneal cavity. The abdominal exudate was centrifuged at 400 g for 10 minutes. The supernatant was discarded and the pellet was washed by PBS twice, and resuspended in 0.2 ml DMEM, the cells were counted. The macrophages isolated from the peritoneal cavity were plated separately in 6-well tissue culture plates and cultured with RPMI/10% fetal bovine serum (FBS) overnight. The following morning, nonadherent cells were removed by aspiration and washing three times with PBS. [3, 21, 22]

### Cell culture

Human umbilical vein endothelial cells (HUVECs, ATCC, Manassas, VA) were cultured in DMEM medium supplemented with 10% FBS. The cells were cultured at 37°C, 95% humidity, and 5% CO_2_. All experiments were performed using cells between passages 4 and 12. The cells were seeded in six-well plates (5 x 10^5^ cells/well), and starved in serum free DMEM medium for 24 hours before the experiments were performed. The macrophages (about 10^6^ cells) isolated from the peritoneal cavity were cultured in RPMI/10% FBS for 3 days. The conditioned medium was collected, centrifuged, filtered, and stored at -20 C in aliquots. The remaining macrophages were collected for real-time PCR experiments. The macrophage-conditioned medium was added to HUVECs in final concentration of 20%. For neutralizing experiments, macrophage-conditioned medium was preincubated with neutralizing TNFα antibody (5 μg/ml) for 1 hour. The conditioned medium was then added to HUVECs to incubate for another 24 hours. Following precipitation of the antigen antibody complex, the concentration of TNFα in the media was measured by ELISA to determine efficacy of neutralization.

### Detection of superoxide anion (O_2_^–^) generation in the aorta

The O_2_^–^ generation in fresh aortic rings was determined by chemiluminescence of lucigenin (5 μmol/L) with Krebs’ buffer, as previously described[23]. The results were expressed as counts/min/mg dry tissue. Chemiluminescence of lucigenin has been validated as method to measure O_2_^-^[23].

### ELISA assay

Plasma levels of C-reactive protein (CRP) and insulin, and TNFα concentration in plasma and conditional media were determined using ELISA assay kits (R&D systems, Minneapolis, Minnesota, USA), following the manufacturer’s instructions, as described in our previous studies[23]. The concentrations were calculated from a standard curve. HOMA-IR, an index for insulin resistance, was calculated by multiple of fast plasma level of glucose by fast plasma level of insulin. The plasma level of cotinine, a stable metabolite of nicotine was also determined using Cotinine ELISA kit (Cal Biotech) following the manuscript’s instruction.

### Organ chamber experiments

Endothelial function in the aortic rings was examined using an organ bath chamber, as previously described[23]. Endothelium-dependent relaxation (EDR) to acetylcholine (10^−9^ to 10^−5^ mol/L) was studied in the rings pre-contracted to 70% of maximal contraction to norepinephrine. Relaxation of aortic rings was expressed as a percentage inhibition of norepinephrine-induced constriction. The maximal response to acetylcholine (Emax) and the concentration of acetylcholine required for a half-maximal response curve (ED_50_) were determined from the concentration-response curve, using best fit to a logistic sigmoid function.

### Western blot

Western blotting analysis was used to determine the protein expression of TNFα and eNOS in the aorta. Briefly, the aorta was cut into small pieces with a scissor, the aortic tissues were placed in a 2 ml via with 3 time volumes of homogenization buffer, the aortic tissues were homogenized for 45 second with 30 second remission at 4 ^o^C, and the homogenization was repeated three times. After the homogenization, the protein content of the different samples was determined by Bio-Rad protein assay (Life Science, Hercules, CA). Thirty μg of protein was separated by SDS-PAGE and transferred to a nitrocellulose membrane. Transferred proteins were incubated overnight with specific primary antibodies against TNFα (1:500 dilution, Santa Cruz Biotech, Santa Cruz, CA) and eNOS (1:1000 dilution, Cell Signaling, Danvers, MA). After washing, the blots were incubated with the appropriate secondary antibody and signal detected by luminal chemiluminescence, followed by exposure to an autoradiography film. The membrane was reblotted for β-actin (1:500 dilution, Santa Cruz Biotech, Santa Cruz, CA) as a loading control.

### Real-time PCR

HUVECs or the macrophages from the peritoneal cavity were harvested in 1 ml trizol reagent, the aortic tissues were homogenated in 1 ml trizol reagent. Total RNA (2 μg) reverse-transcribed using a superscript II RT first strand synthesis kit (Gibco, BRL) according to the manufacturer’s instructions. Real-time PCR was performed with a TaqMan master mix assay kit (ABI). Relative quantities of each transcript were normalized by a housekeeping gene (GAPDH) and expressed as fold increase vs. control. All PCR primers with fluorescence probe for CD36 (assay ID: Rn00590726-m1), TNFα (Rn09999017-μ1, interleukin (IL) 1β (Rn01336189-m1), eNOS (Rn02132634-s1), gp91phox (Rn00576710-m1), p22phox (Rn00577357-m1) were ordered through ABI online system with assay IDs.

### PCR array for determination of mRNA profile of rat inflammatory signaling pathway

The aortic tissues were homogenated in 1 ml trizol reagent, one μg of RNA was converted to cDNA with random primers in a 20-μl reaction volumes using high capacity cDNA archive kit (C-3, Superarray). The cDNA was diluted in total volume 100 μl. One μl of cDNA was used for each primer set in the PCR array according to the manufacturer’s instruction as previously described[24]. Rat inflammatory cytokine & receptor signaling pathway was used to determine a panel of inflammatory gene expression (PARN-011Z, SuperArray Qiagen). This PCR array includes 84 target genes of inflammatory chemokines, cytokines, interleukins and their receptors and 12 control genes. The control genes include each array for genomic DNA contamination, RNA quality, housekeep and general PCR performance. Data analysis was performed using the manufacturer’s integrated web-based software package for the PCR array system using delta-delta Ct based fold-change calculations and normalized by a housekeeping control gene.

### Data analysis

The results were expressed as means ± SE. Statistical analyses for all parameters were performed by two way ANOVA followed with Bonferonni’s correction for multiple comparisons (StatView, BrainPower, Calabasas, CA). Significance was assumed at P< 0.05.

## Results

### Effects of nicotine on SBP, body weight, plasma level of cotinine and metabolic variables in diet-induced obese rats

**Fig 1:**
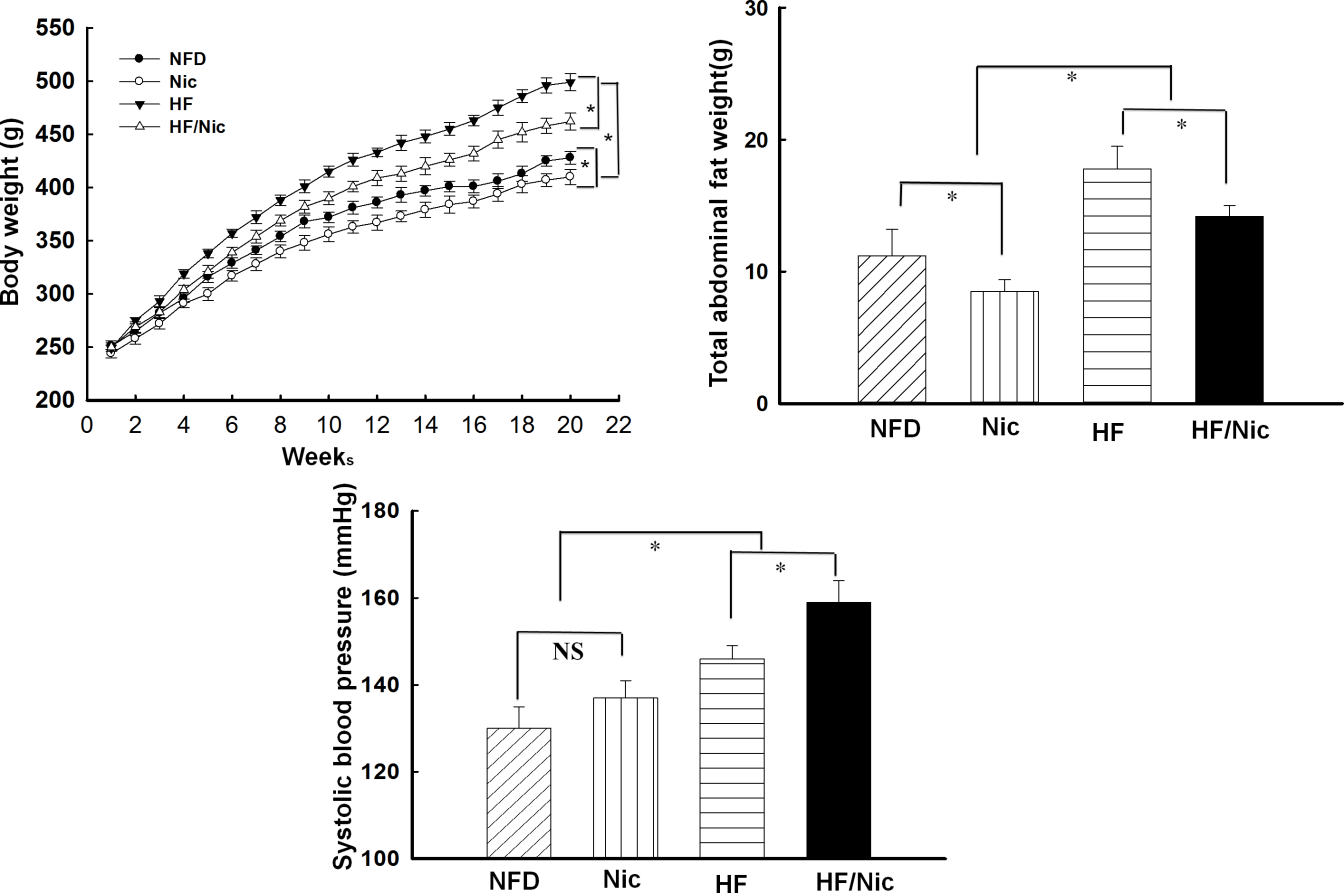
**Body weight (A), total abdominal fat weight (B) and systolic blood pressure (SBP, C) in nicotine-treated obese rats.** The data was expressed as mean ± SE. NFD: normal rat chow diet; HFD: high fat diet; Nic: NFD with nicotine; HFD/Nic: HFD with nicotine treatment. N = 6–7, *p<0.05.

**Fig 2:**
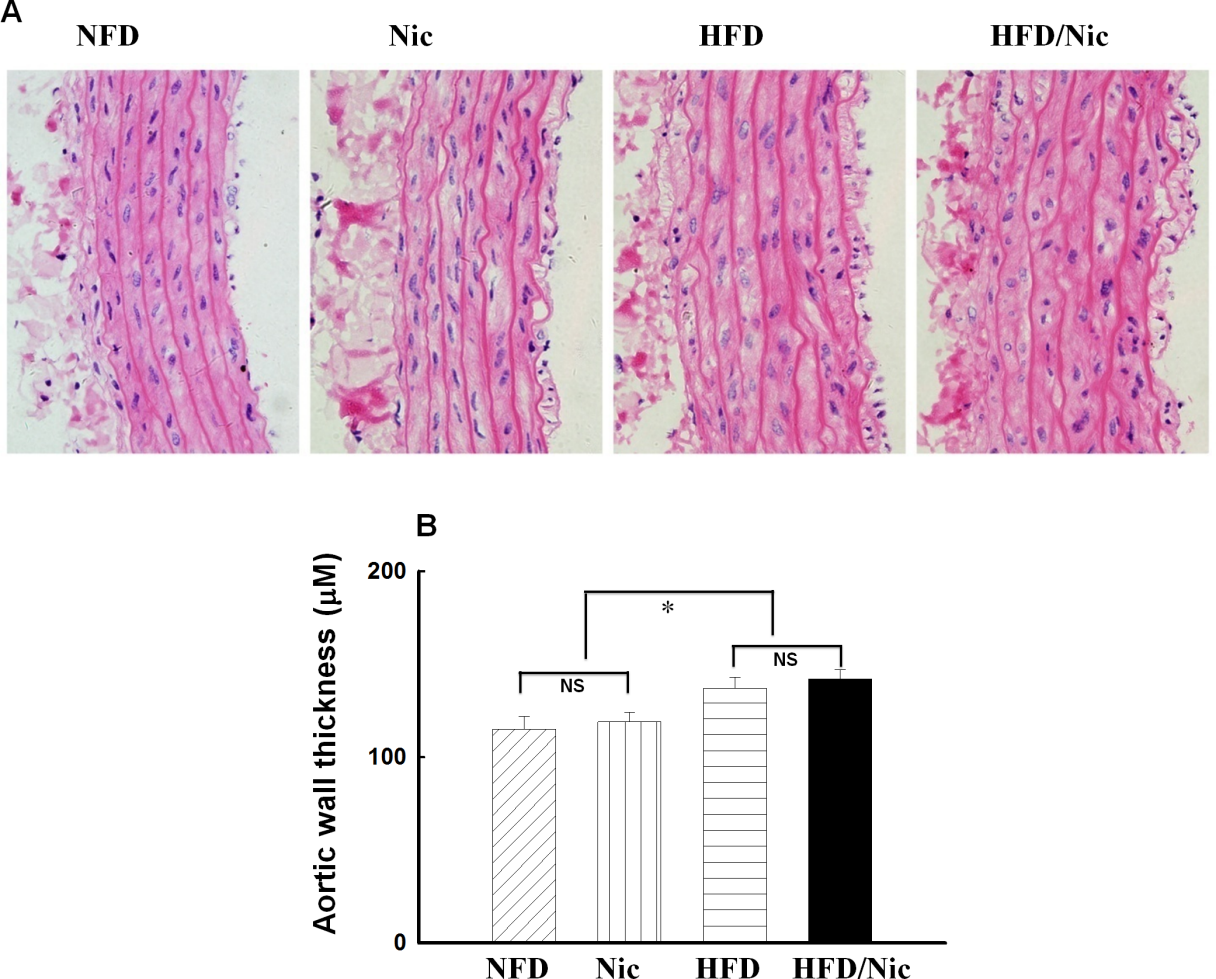
Histological examination of aortic hypertrophy in nicotine treated obese rats. Representative cross section of aortic wall (A) stained with HE. Bar graphs show the quantitative analysis of aortic wall thickness (B). *p< 0.05 vs. NFD, *N* = 6–7.

HFD for 20 weeks increased SBP (146 ± 4 vs. 131 ± 5 mmHg in NFD, p<0.05), compared with NFD rats. Nicotine treatment further increased SBP in obese rats (159 ± 5 vs. 146± 4 mmHg in obese rat, p<0.05), and had a tendency to increase SBP in lean rats but did not reach a statistical significance. There were no statistically significant differences in SBP between NFD and Nic group (131 ± 5 mmHg in NFD vs. 138 ± 4 mmHg in Nic, p>0.05). Compared with the NFD group, HFD for 20 weeks resulted in significant increase in body weight (495 ± 10 vs. 432 ± 8 g in NFD, 14.5% increase, p<0.05) and total abdominal fat weight (17.8 ± 1.7 vs. 11.6 ± 2.3 g in NFD, 53% increase, p<0.05). Nicotine significantly inhibited body weight gain and abdominal fat weight in both NFD and HFD rats (all p<0.05, Fig 1). HFD for 20 weeks significantly increased fast plasma levels of glucose, insulin, cholesterol, NFFA and HOMA-IR (Table 1), the rats on HFD diet developed central obesity with insulin resistance. Long-term treatment with nicotine did not affect above metabolic variables (Table 1) in either NFD or HFD rats. The plasma levels of cotinine, a stable metabolite of nicotine, were undetectable in both control and obese rats without nicotine treatment, and were increased by around 5–10 ng/ml in the rats receiving oral administration of nicotine (Table 1). The plasma concentration of cotinine in the rats with nicotine treatment is correlated with plasma cotinine levels found in secondhand smokers and nicotine patch user in human[25, 26]. HFD significantly increased aortic weight but did not affect the ratio of heart weight/body weight. Nicotine treatment did not affect aortic weight and the ratio of heart weight/body weight in NFD and HFD rats (Table 1). Histological study revealed that diet-induced obese rats (HFD) exhibited a significant aortic medial growth and increased aortic wall thickness. In NFD and HFD rats, nicotine treatment had no effects upon aortic medial growth and aortic wall thickness (Fig 2).

**Table 1:** Effects of nicotine and obesity on metabolic parameters.

	NFD	Nic	HFD	HFD/nic
**HW (g/100 g BW)**	**0.31 ± 0.01**	**0.31 ±0.01**	**0.3 ± 0.01**	**0.3 ± 0.01**
**AW (mg/cm)**	**17.4 ± 0.5**	**17.0 ± 0.5**^**†**^	18.7 ± 0.4^*^	18.6 ±0.7^*^
**Plasma glucose** **(mg/dl)**	**112 ± 5**	**113 ± 4**^**†**^	125 ± 2^*^	124 ± 3^*^
**Plasma insulin** **(ng/ml)**	**3.3 ±0.2**	**3.4 ± 0.2**^**†**^	3.8 ±0.1^*^	**3.8 ± 0.1**
**Plasma cotinine** **(ng/ml)**	**undetectable**	**5.8 ± 1.6**	**Undetectable**	**9.1 ± 2.8**
**Plasma cholesterol** **(mg/dl)**	**83 ± 5**	**79 ± 3**^**†**^	112 ± 4^*^	106 ± 7^*^
**NFFA (mmol/L)**	**0.60 ± 0.04**	**0.68 ±0.1**^**†**^	1.07 ±0.21^*^	0.99 ±0.06^*^
**HOMA-IR**	**24.6 ± 1.4**	**25.6 ± 1.1**^**†**^	31.7 ± 1.3^*^	31.4 ± 1.5^*^

NFD: low fat diet; HFD: high fat diet; Nic: nicotine; HW: heart weight; AW: aortic weight; BW: body weight; NFFA: noesterified free fatty acids. Data was expressed as mean ± SE,

*p<0.05, vs. NFD;

^†^p<0.05, vs. HFD. N = 5–6.

### Effects of nicotine on aortic O_2_^-^production and plasma CRP in diet-induced obese rats

**Fig 3:**
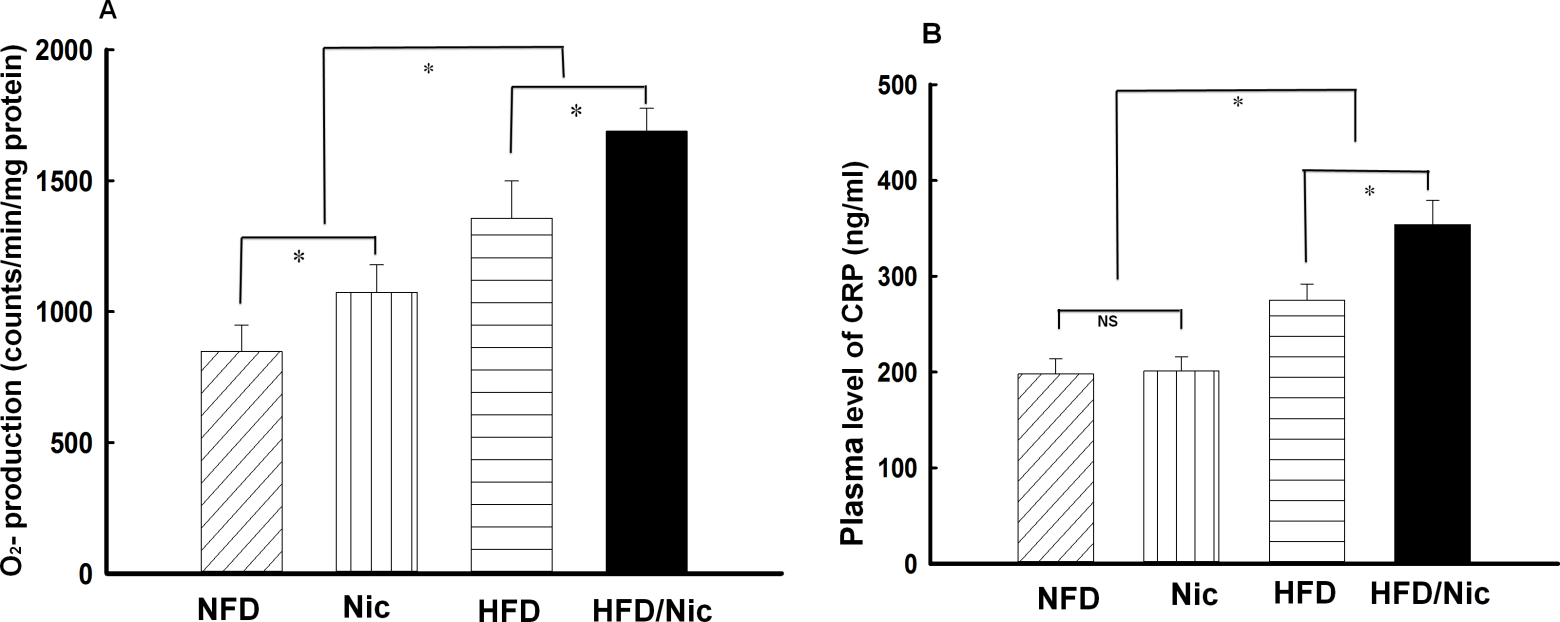
**Aortic superoxide (O**_**2**_^**-**^**) production (A) and plasma level of C-reactive protein (CRP, B) in nicotine-treated obese rats.** HFD for 20 weeks increased aortic O_2_^-^ production and plasma level of CRP. Nicotine increased O_2_^-^ production in both lean and obese rats, and increased plasma level of CRP only in HFD rats. *p<0.05, vs. NFD, #p<0.05 vs. HFD, N = 6–7.

Increased ROS production has been implicated in the pathogenesis of metabolic syndrome[27]. We have previously shown that stimulation of nicotinic acetylcholine receptor with nicotine increased ROS production via NADPH oxidase[3, 28]. Here we examined the effect of nicotine on aortic O_2_^-^ production in diet-induced obese rats. As shown in Fig 3A, aortic O_2_^-^ production was significantly increased in diet-induced obese rats. Nicotine increased O_2_^-^ production in lean rats and further increased O_2_^-^ production in diet-induced obese rats, suggesting that nicotine and obesity additively increased vascular O_2_^-^ production. CRP is an acute phase reactant, and increased plasma CRP is a reliable biomarker to predict for development of CVD. As shown in Fig 3B, plasma level of CRP was significantly increased in diet-induced obese rats. Nicotine treatment further increased plasma level of CRP in obese rats but not in lean rats (Fig 3B), suggesting that nicotine increased systemic inflammation in obese rats.

### Nicotine increased the mRNA expressions of CD36, TNFα and IL1β in the peritoneal macrophages of diet-induced obese rats

**Fig 4:**
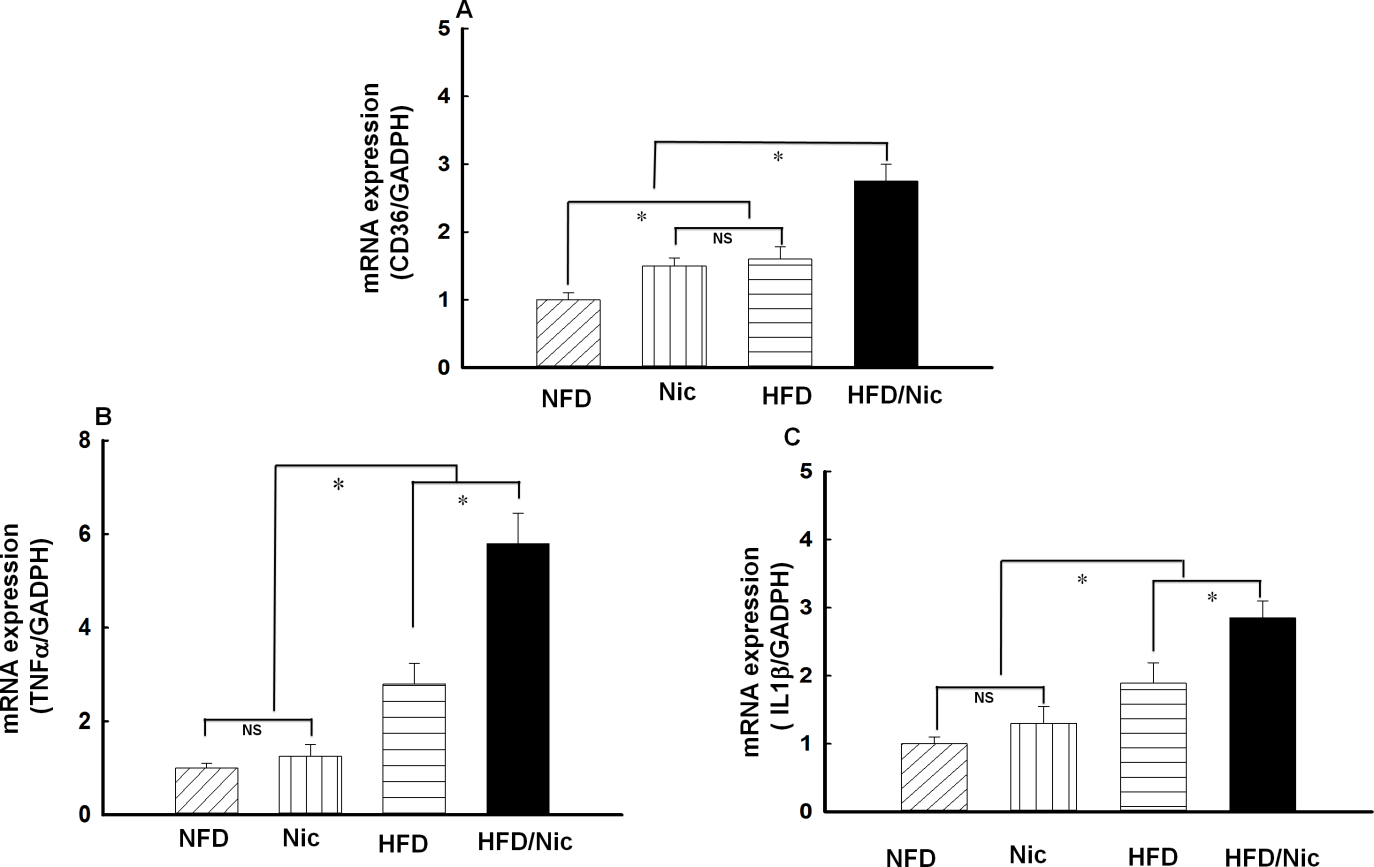
**The mRNA expressions of CD36 (A), TNFα (B) and IL1β (C) in the peritoneal macrophages of nicotine-treated obese rats.** The mRNA expressions of TNFα, IL1β and CD36 were significantly increased in the peritoneal macrophages of diet-induced obese rats. Nicotine further increased the mRNA expressions of three molecules in obese (HFD) rats and the mRNA expression of CD36 in lean rats. N = 5, *p<0.05, vs. NFD, #p<0.05, vs. HFD.

CD36 is the major scavenger receptor for the uptake of oxLDL in macrophages[29]. We have recently shown[3] that nicotine promotes foam cell formation and atherosclerosis via upregulating macrophage CD36 signaling. As shown in Fig 4A, the mRNA expression of CD36 was significantly increased in the peritoneal macrophages of diet-induced obese rats and was further increased in nicotine-treated obese rats. Proinflammatory cytokines such as TNFα and IL1β are important markers of activated inflammatory macrophage (M1). As shown in Fig 4B and 4C, the mRNA expressions of TNFα and IL1β were significantly increased in the peritoneal macrophages of diet-induced obese rats. Nicotine further potentiated the expressions of those inflammatory genes in obese rats.

### Nicotine and obesity additively/synergistically increased proinflammatory gene expression in the aorta

It has been generally accepted that obesity is associated with chronic low-grade inflammation[17, 30]. We have recently shown that nicotine promoted vascular inflammation and atherosclerotic lesion formation in apo E^-/-^ mice[3]. Here we used PCR array of rat proinflammatory signaling pathway to determine a panel (84 target genes) of proinflammatory gene expression in the aorta. This PCR array includes genes of inflammatory chemokines, cytokines, and interleukins. As shown in Table 2, among the 84 target genes, 4 genes including MCP1, interferon γ, TNFα and chemokine (C-X-C motif) ligand (CXCL)11 were increased by approximately 2–4 folds in diet-induced obese rats; 11 genes, including MCP1, interferon γ, TNFα, CXCL11, CXCL10, CXCL9, IL4, IL6, CXC chemokine receptor 3, macrophage migration inhibitor factor and CD14 ligand, were increased by approximately 2–10 folds in nicotine-treated obese rats as compared with NFD rats. Nicotine further increased 10 of 11 genes expressions (except MCP1) in diet-induced obese rats (Table 2), but not in lean rats. These results suggest that nicotine and obesity additively/synergistically promoted vascular inflammation.

**Table 2:** mRNA profile of inflammatory cytokines in the aorta of nicotine-treated obese rats (data was normalized by NFD group as 1, determined by PCR array).

	NFD	Nic	HFD	HFD/Nic
**CCL2 (MCP1)**	**1 ± 0.13**	**1.03 ± 0.12**	2.13 ± 0.25^*^	2.01 ± 0.26^*^
**MIF**	**1 ± 0.15**	**0.97 ± 0.20**	**1.21 ± 0.42**	3.01 ±0.75^*^^#^
**CXCL9**	**1 ± 0.25**	**0.92 ± 0.22**	**1.25 ± 0.45**	5.54 ± 1.25^*^^#^
**CXCL10**	**1 ± 0.35**	**0.77 ± 0.28**	**1.34 ± 0.25**	2.76 ±0.80^*^^#^
**CXCL11**	**1 ± 0.23**	**0.85 ± 0.15**	2.79 ± 0.55^*^	7.51 ± 1.32^*^^#^
**CXCr 3**	**1 ± 0.18**	**0.73 ± 0.22**	**0.83 ± 0.30**	3.26 ± 1.12^*^^#^
**Interferon γ**	**1 ± 0.20**	**0.92 ± 0.35**	3.92 ± 1.02^*^	9.82 ± 2.15^*^^#^
**IL4**	**1 ± 0.22**	**1.09 ± 0.16**	**1.35 ± 0.50**	3.63 ± 0.65^*^^#^
**IL6**	**1 ± 0.10**	**1.21 ± 0.15**	**1.25 ± 0.22**	5.45 ± 0.72^*^^#^
**TNFα**	**1 ± 0.35**	**1.39 ± 0.37**	3.52 ± 0.65^*^	5.25 ± 0.86^*^^#^
**CD40 ligands**	**1 ± 0.12**	**1.13 ± 0.22**	**1.45 ± 0.33**	3.43 ± 0.98^*^^#^

Nic: nicotine, MCP-1: monocyte chemoattract protein 1, MIF: macrophage migration inhibitor factor, TNFα: tumor necrosis factor α, IL: interleukin. Data was normalized and expressed as fold increase by lean group. Data was expressed as mean ± SE, 4 PCR array for each group.

*p<0.05 vs. NFD,

^#^p<0.05 vs. HFD.

### Nicotine increased TNFα and reduced eNOS expression in the aorta of diet-induced obese rats

**Fig 5:**
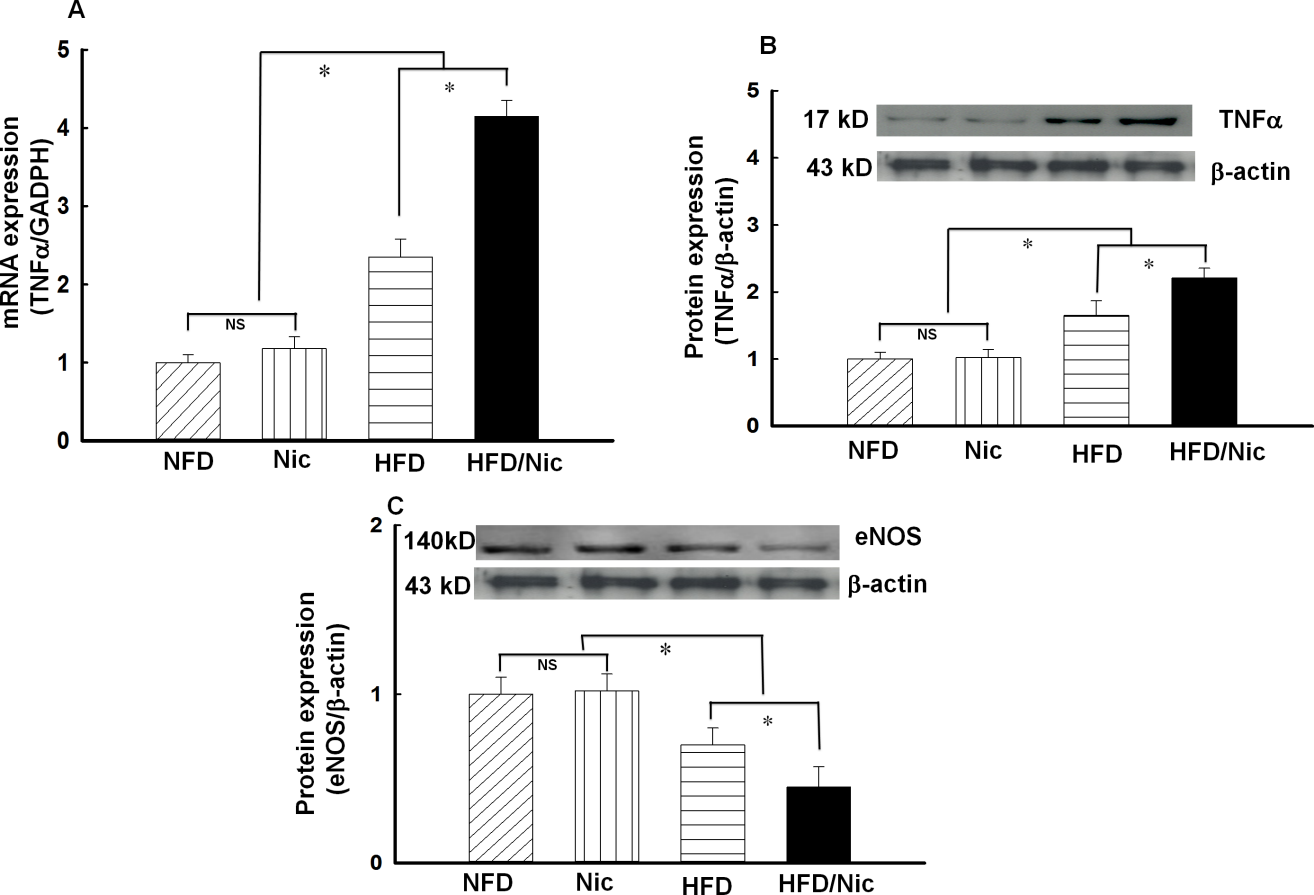
**The mRNA (A) and protein (B) expressions of TNFα or protein expression of eNOS (C) in the aorta of nicotine-treated obese rat.** N = 5, *p<0.05, vs. NFD, #p<0.05 vs. HFD.

TNFα is an important inflammatory cytokine released from activated macrophage, T cells, endothelial cells and adipocytes, and has been shown to induce macrophage activation and endothelial dysfunction[31, 32]. As shown in Fig 5A and 5B, the mRNA and protein expressions of TNFα were increased in the aorta of obese rats and further increased in nicotine-treated obese rats. Reduction in eNOS-derived NO production is considered a hallmark of endothelial dysfunction. As shown in Fig 5C, the protein expression of aortic eNOS was significantly reduced in obese rats. Nicotine further decreased aortic eNOS expression in obese rats, suggesting that nicotine and obesity have a synergistic reduction in eNOS expression.

### Nicotine impaired EDR to acetylcholine in the aorta of obese rats

**Fig 6:**
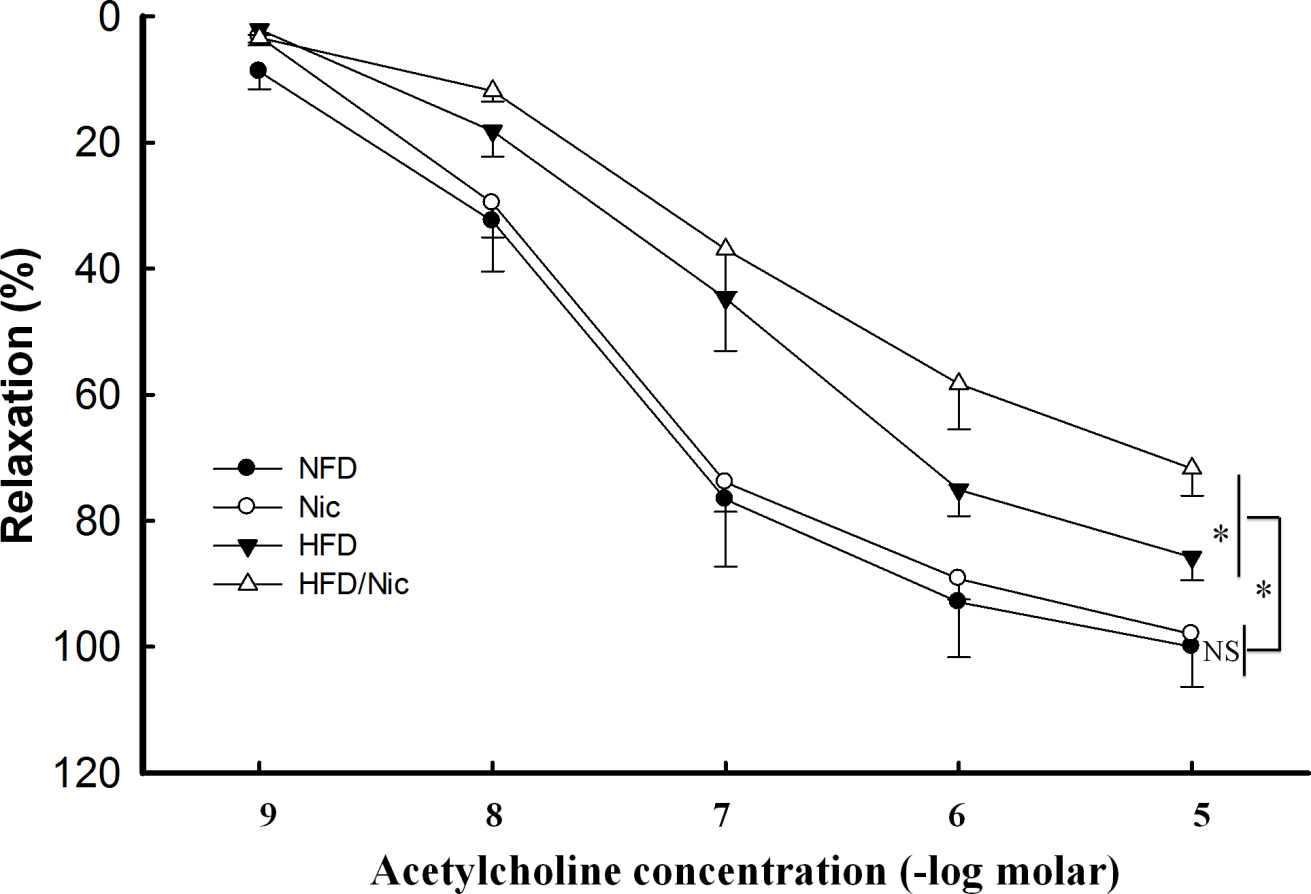
Endothelium-dependent relaxation (EDR) to acetylcholine in the aorta of nicotine-treated obese rats. EDR to acetylcholine was attenuated in obese rats. Nicotine further impaired the EDR to acetylcholine in obese rat but not in lean rats. NS: no significant difference. N = 5–7, *p<0.05.

Endothelial dysfunction, characterized by loss of endothelium-dependent relaxation, is thought to be a key event in the development of atherosclerosis and other vascular diseases[33]. As shown in Fig 6, EDR to acetylcholine was significantly attenuated in the aorta of diet-induced obese rats (Emax: 85 ±6% in HFD rats vs. 98 ± 3% in NFD rats, p<0.05; ED_50_: 6.9 ±0.1 vs. 7.1 ± 0.1 -log molar in NFD rats, p<0.05), compared with NFD rats. Nicotine further impaired EDR to acetylcholine in obese rats (Emax: 85 ± 6% in HFD rats vs. 72 ± 5% in the HFD/Nic rats, p<0.05; ED_50_: 6.9 ±0.1 vs. 6.6 ± 0.1 -log molar in NFD/Nic rats, p<0.05) but not in lean rats (Fig 6).

### Effects of the macrophage conditioned medium on the mRNA expressions of eNOS, gp91phox and p22phox in HUVECs

**Fig 7:**
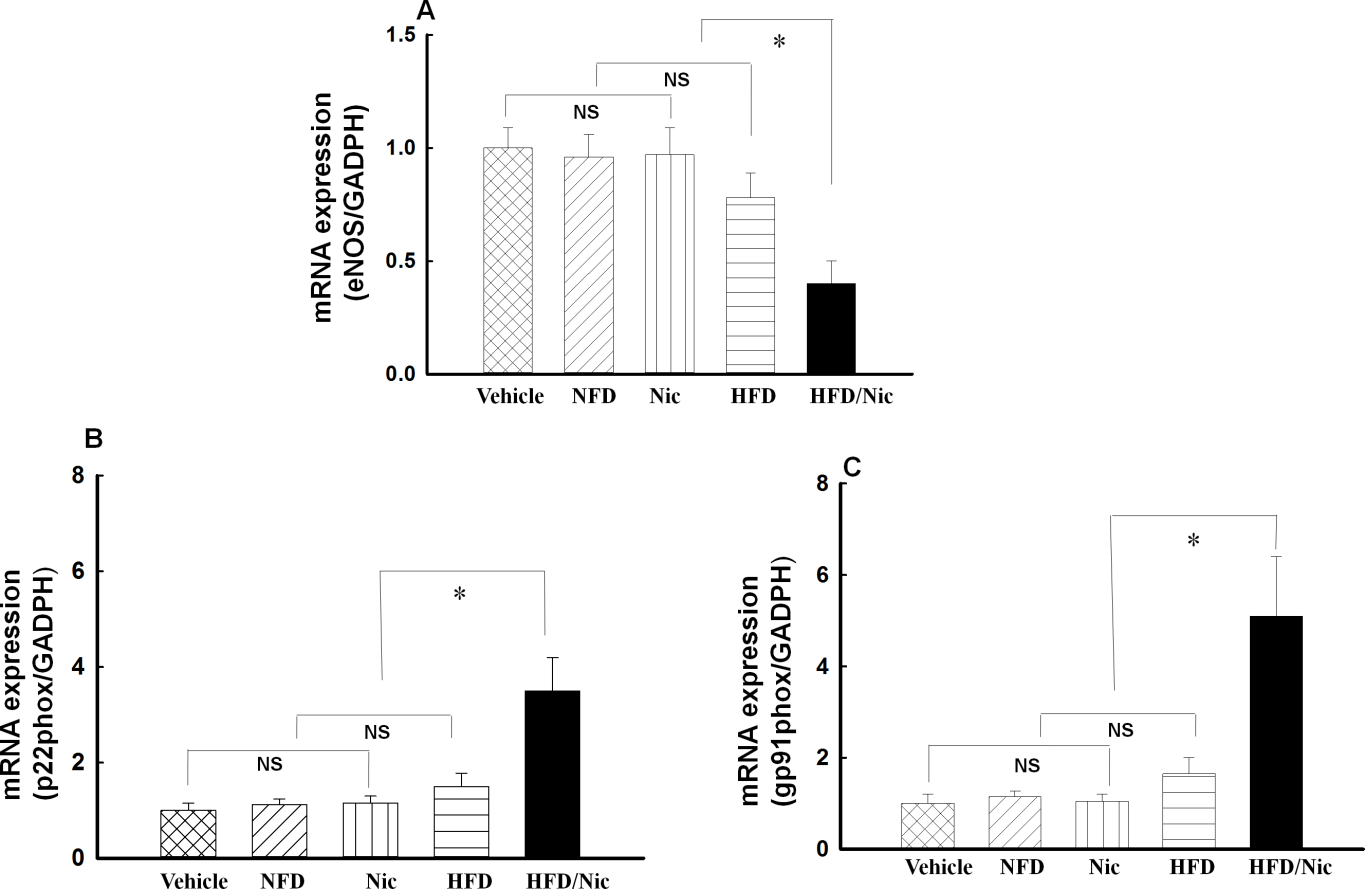
**Effects of the macrophage conditioned medium on the mRNA expressions of eNOS (A) and NADPH oxidase subunits gp91phox (B) and p22phox (C) in HUVECs.** *p<0.05, NS: no significant difference. N = 5.

HUVECs were pre-incubated with vehicle medium or the conditioned medium (20%) from the peritoneal macrophages for 24 hours, HUVECs were collected for determination of eNOS and NADPH oxidase subunits gp91phox and p22phox mRNA expression. As shown in Fig 7, incubation with the conditioned medium from lean (NFD), nicotine-treated lean (Nic) or HFD groups did not significantly affect the mRNA expressions of eNOS, gp91phox or p22phox in HUVECs, as compared with vehicle medium. Only incubation with the conditioned medium from nicotine-treated obese rats resulted in a significant reduction in the mRNA expression of eNOS or increase in the mRNA expressions of gp91phox and p22phox in HUVECs (all p<0.05, Fig 7B and 7C).

### TNFα antibody partially restored the expressions of eNOS, gp91phox and p22phox in HUVECs induced by the conditioned medium

**Fig 8:**
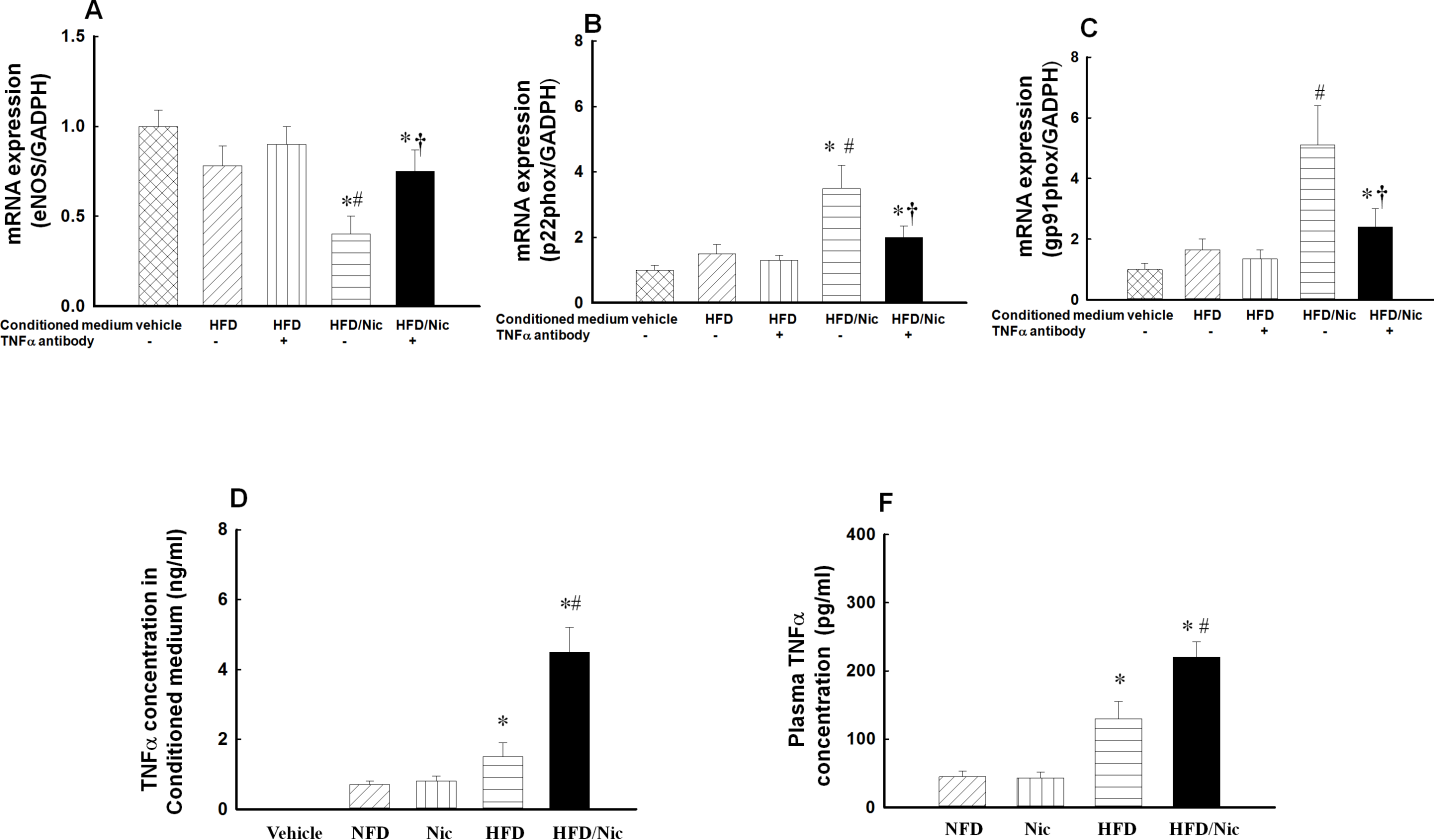
**Effects of neutralizing TNFα antibody on the mRNA expressions of eNOS (A), gp91phox (B) and p22phox (C) in HUVECs.** The conditioned medium was pre-incubated with neutralizing TNFα antibody for one hour to block TNFα effects, the medium then was added to HUVECs to incubate for another 24 hours, the mRNA expressions of eNOS, gp91phox and p22phox in HUVECs were determined by real-time PCR. TNFα antibody in the conditioned medium from nicotine-obese rats partially restored the expression of eNOS, gp91phox, p22phox. TNFα concentration in conditioned medium (D) or in plasma (F) was increased in obese (HFD) rats, and was further increased in nicotine-obese rats. *p<0.05 vs. vehicle medium; #p<0.05 vs. HFD; †p<0.05 vs. correspondence group without TNFα antibody. N = 5–6.

To determine the role of TNFα in the macrophage conditioned medium-induced eNOS and NADPH oxidase dysfunction, the conditioned medium from the peritoneal macrophages were preincubated with neutralizing TNFα antibody (5 μg/ml) for 1 hour. The conditioned medium was then added to HUVECs in final concentration of 20% and incubated for another 24 hours. As shown in Fig 8, TNFα antibody in the conditioned medium from HFD rats did not significantly affect the mRNA expressions of eNOS, gp91phox or p22phox in HUVECs, but TNFαantibody in the conditioned medium from nicotine-obese rats in part prevented a decrease in eNOS and an increase in gp91phox and p22phox expression in HUVECs, as compared with the correspondence conditioned medium without TNFα antibody. Furthermore, we measured TNFα concentration in plasma and the conditioned medium by ELISA, TNFα concentration was 0.7 ± 0.1 ng/ml in the conditioned medium from lean rats, 0.8 ± 0.2 ng/ml in nicotine-lean rats, and 1.5 ± 0.4 ng/ml in obese rats, 4.5 ± 0.7 ng/ml in nicotine-obese rats. TNFα was undetectable in the vehicle medium or the conditioned medium treated with neutralizing TNFα antibody (Fig 8D). Plasma level of TNFα was significantly increased in obese rats compared with lean rats, nicotine treatment further increased plasma level of TNFα in obese rats, but not in lean rats (Fig 8E). The results suggest that nicotine further increases macrophage production/release of TNFα in obese rats but not in lean rats. TNFα may in turn induce endothelial dysfunction via disrupting the balance between vascular eNOS and ROS.

## Discussion

The major findings of this study are that long-term oral treatment with nicotine augmented endothelial dysfunction, vascular oxidative stress and vascular inflammation in diet-induced obese rats, which were associated with the enhancement of the macrophage release of proinflammatory cytokines. Most of those effects except oxidative stress, however, were not seen in nicotine-treated lean rats. Furthermore, treatment with the conditioned medium from the peritoneal macrophages of nicotine-obese rats inhibited eNOS expression and increased NADPH oxidase subunits gp91phox and p22phox expressions in HUVECs, which were in part prevented by addition of neutralizing anti-TNFα antibody to the conditioned medium.

Cigarette smoke is a well-established risk factor for CVD and atherosclerosis[34]. The health care risk associated cigarette smoke for CVD is exaggerated by obesity or type II diabetic mellitus[6, 19, 20]. Nicotine has been suggested to participate in many of the adverse effects of smoke on the CV system[7], although there are also opposite reports that chronic nicotine treatment may have some beneficial vascular effects[35] We and others[3, 36] have shown that chronic nicotine treatment promotes atherosclerotic lesion formation in apo E^-/-^ mice. Nicotine replacement therapy (NRT) is widely used for smoking cessation[37]. NRT for a short term is considered as relatively safe, although long term NRT regarding CV safety has not been determined[37]. The present study showed that long-term treatment with nicotine (20 weeks) did not cause vascular structure and functional damage in normal rats. However, nicotine treatment did augment endothelial dysfunction, vascular oxidative stress and inflammation in diet-induced obese rats. Importantly, our results shows that plasma levels of cotinine in nicotine-treated rats are about 5–10 ng/ml, which is comparable with plasma level in nicotine patch users in human[26]. These results suggest that long term NRT may be less toxic than cigarette smoking but it may also not be fully safe, particularly for the those who are obese, have type 2 diabetes or concomitant with CV risk factors such as hypertension.

Multiple mechanisms have been suggested as the underlying link between nicotine and CVD, including inflammation, endothelial dysfunction/injury, activation of thrombosis, and the modification of the lipid profile[34, 38]. The pro-inflammatory or anti-inflammatory effects of nicotine have been the subjects of much controversy[36, 39, 40]. Nicotine has been often implicated as anti-inflammatory[40, 41]. Acting through nicotinic acetylcholine receptors on neurons, nicotine can have anti-inflammatory effects protecting, for example, against neural damage during inflammation associated with Parkinson's disease or traumatic brain injury[42]. On the other side, nicotine was also found to be a strong proinflammatory mediator[43]. We and others have shown that nicotine stimulates monocytes/macrophages-release inflammatory cytokines, and promotes monocyte/macrophage adhesion to endothelium and migration into vascular wall, resulting in vascular inflammation and acceleration of atherosclerotic process in vitro and in vivo [3, 36, 44].

Our results from PCR array showed that nicotine had different effects on the expression of proinflammatory cytokines in the aorta of obese and lean rats, increased the mRNA expressions of inflammatory cytokines in obese rats but not in lean rats. Harwani et al[45] reported that nicotine increased plasma levels of proinflammatory cytokines in prehypertensive spontaneously rats but suppressed those responses in Wistar Kyoto rats, suggesting an opposite response of nicotine on inflammatory system in those two strains of rats. The mechanisms why nicotine exhibits a different proinflammatory response in lean and obese rats are not investigated in this study. We have recently shown that nicotine promotes macrophage activation and release of inflammatory cytokines by upregulating CD36 inflammatory pathway[3]. oxLDL (a CD36 ligand) is required for nicotine activation of this pathway[3]. Here we showed that nicotine and obesity additively upregulated macrophage CD36 expression. Obesity is associated with oxidative stress and increased oxLDL[46, 47], therefore it is reasonable to speculate that the upregulation of macrophage CD36 may interact with oxLDLs to initiate inflammatory responses in nicotine-treated obese rats, but the similar pathway may not be activated by nicotine in lean rats because there may not have enough oxidized LDL products to stimulate CD36 inflammatory pathway in lean rats.

Nicotine can induce endothelial dysfunction[48, 49]. Acute and chronic administration of nicotine impairs endothelium-dependent relaxation and produces morphological abnormalities of endothelium[7, 50]. Endothelial dysfunction represents a key step in the initiation and maintenance of atherosclerosis and may be a marker for further risk of cardiovascular diseases. Reduced NO bioavailability caused by oxidative stress is a common feature of endothelial dysfunction[51, 52]. Here we showed that nicotine augmented endothelial dysfunction accompanied with further increased oxidative stress and decreased eNOS expression in the vasculature of diet-induced obese rats. Therefore, we surmise that nicotine may augment endothelial dysfunction via further induction of imbalance between vascular NO and ROS.

Inflammatory cytokines can target vascular endothelium to induce endothelial dysfunction[53], for example, TNFα has been shown to induce endothelial dysfunction associated with increased oxidative stress and decreased eNOS expression[13, 31]. TNFα is mainly produced in immune cells such as macrophages. To investigate the role of macrophage TNFα in nicotine induction of endothelial dysfunction in obese rats, we examined the effects of macrophage conditioned medium on eNOS and NADPH oxidase subunits gp91phox and p22phox in HUVECs. Here we found that TNFα concentration/expression was increased in the plasma, the culture medium, the peritoneal macrophages and the aorta from nicotine-treated obese rats. HUVECs, treated with the macrophage conditioned medium from nicotine-treated obese rats, exhibited a significant decrease in eNOS expression and increase in the expressions of gp91phox and p22phox. Those effects were partially reversed by adding anti-TNFα antibody into the conditioned medium. Based on those results, we speculate that nicotine induces endothelial dysfunction at least in part through promoting the macrophage production/release of TNFα in obese rats, which result in disrupting the balance between eNOS and ROS in the endothelium.

It is well known that nicotine reduces body weight[54, 55]. Consistent with previous studies[54], we observed that long-term treatment with nicotine reduced body weight and abdominal fat weight in both lean and obese rats. The effect of nicotine on reducing body weight was more pronounced in obese rats than in lean rats. Our data also showed that nicotine increased blood pressure in obese rats. Clinically, increased BP by nicotine may also increase CV risk in obesity.

In conclusion, the present study provides convincing evidences that nicotine aggravates endothelial dysfunction and vascular inflammation with cellular oxidative stress and impaired eNOS in diet-induced obese rats. Those deteriorating vascular effects of nicotine can be in part explained by enhancement of macrophage-derived TNFα, which may target endothelium. More importantly, plasma levels of cotinine in nicotine-treated rats are comparable with the plasma cotinine levels found in secondhand smokers, nicotine patch users or individuals with chronic inhalation of nicotine such as e-cigarette[25, 26, 56], those subjects, after exposure to nicotine, may increase the risk for vascular atherosclerotic diseases, particularly in the patients with obesity or type II diabetes. Additional research in cellular and molecular studies should enable the determination of important mechanistic insights in nicotine aggravation of CV risk in obesity.

## Supporting information

S1 File Experimental protocols. (DOC)

S2 File Supporting data file. (XLSX)
