# Supplementary material for: Oral nicotine aggravates endothelial dysfunction and vascular inflammation in diet-induced obese rats: Role of macrophage TNFα
Source: PLoS One. 2017 Dec 13;12(12):e0188439. doi: 10.1371/journal.pone.0188439 (PMC5728478; doi:10.1371/journal.pone.0188439)
Supplement: S1 File — (DOC) [file pone.0188439.s001.doc]

**Supplemental Materials (S1): Experimental Protocols**

**Oral Nicotine Aggravates Endothelial Dysfunction and Vascular Inflammation in Diet-induced Obese Rats: Role of Macrophage TNFα**

Chang Liu^1^, Ming-Sheng Zhou^2,3^, Yao Li^3^, Aimei Wang^3^, Kiranmai Chadipiralla^4^, Runxia Tian^4^, Leopoldo Raij^4^

1 Department of Endocrinology, First Affiliated Hospital, Jinzhou Medical University; 2 Department of Physiology, Shenyang Medical University, Shenyang, Liaoning, 3 Department of Physiology, Jinzhou Medical University, Jinzhou, Liaoning, P.R. of China; 4 Nephrology-Hypertension Section, University of Miami Miller School of Medicine, Miami VAMC, Miami, USA.

**Animals and experimental protocols:**

**Protocol information:**

**ACORP #: 9873.02 (**The Institutional Animal Care and Use Committee at the Miami VA Medical Center)

Diet information:

**N**ormal rat chow diet: Dyets Inc. (Bethlehem, PA ), Cat#: No. 183018 (total kcal/kg: 3605)

High fat rat chow diet: Dyets Inc. (Bethlehem, PA ), Cat#: No. 183025 (total kcl/kg: 4523)

**Procedures:**

The animals were housed in facilities accredited by the American Association for Accreditation of Laboratory Animal Care and by the Chinese Association for Accreditation of Laboratory Animal Care. The Institutional Animal Care and Use Committee at the Miami VA Medical Center (protocol #: 9873.02) and Jinzhou Medical University approved the studies. All procedures were performed in accordance with the Guide for the Care and Use of Laboratory Animals published by the US National Institutes of Health (Eighth Edition, the Guide, NRC 2011). Six-week-old Sprague-Dawley male rats were purchased from Harlan Sprague-Dawley Inco. (Indianapolis, IN) and maintained under controlled conditions of light, temperature, and humidity. After having 2 weeks to accommodate to the new environment, the rats were randomly divided into 4 groups and treated for 20 weeks (n=6-7): NFD (normal fat diet): fed a normal rat chow diet (17% caloric from fat, Cat#:No. 183018, Dyets Inc. Bethlehem, Pa); Nic: fed a NFD diet with nicotine (100 mg/L in drinking water); HFD (high fat diet): fed a high fat diet (47% caloric from fat, containing 20% (wt/wt) hydrogenated coconut oil, Cat#: No. 183025, Dyets Inc.); HFD/Nic: fed a HFD plus nicotine treatment. Body weight was measured every week. Systolic blood pressure (SBP) was measured in the conscious rats by the tail-cuff method. At the end of the study, the rats were starved overnight, fasted plasma glucose was measured by blood glucose meter. Fasted plasma cholesterol and nonesterified free fatty acids (NFFA) were determined by cholesterol assay kit (Wako Diagnostics, Cat #: 435-35801, Richmond, VA) and NFFA assay kit, respectively (Wako Diagnostics, Cat#: 999-34691, Richmond, VA). The rats were anesthetized by sodium pentobarbital (50 mg/kg IP) and euthanized by decapitation; the heart, aortas and total abdominal fat were weighed. The heart weight/body weight and aortic weight/cm (arch of thoracic aorta to the origin of mesenteric artery) were used as indices of cardiac and aortic hypertrophy.

**Systolic blood pressure measurement by tail cuff methods:**

**Instrument:** BP-2000 blood pressure analysis system (Visitech system, NC),

**Procedures:** Systolic blood pressure (SBP) was determined in the conscious mice using the tail cuff method (BP-2000 blood pressure analysis system) as described in our previous study(Zhou et al., 2015). The rats were trained for 5 consecutive days to adapt to SBP measurement before the experiments were performed. The rats were placed in plates in BP-2000 blood pressure operate system and preheated for 15 minutes to dilate tail arteries, SBP was measured in a quiet and dark room. SBP was measured every week. At least 5 successive readings were recorded and averaged for each mouse.

# Histological analysis: The aorta (1 cm below the highest point of the aortic arch) was isolated. The specimens from the thoracic aorta were fixed in 4% paraformaldehyde in phosphate-buffered saline. The 4-μm thick sections were cut and stained with hematoxylin and eosin. Four nonconsecutive digital images per animal were acquired with a LC Evolution camera (Media Cybernetics, Inc., Bethesda, Maryland, USA) and Olympus BX51 microscope (Leeds Precision Instruments, Inc., Minneapolis, Minnesota, USA) and analyzed with Image-Pro Plus version 6.0 software (Media Cybernetics, Inc.). The radial thickness of the media was measured.

**Isolation of peritoneal macrophages:** Another set group of rats as described above were used to isolate the macrophages from the peritoneal cavity, the rats were injected intraperitoneally (I.P) with 5 ml of 3% thyoglycolate (Sigma Aldrich, St. Louis, MO) medium to induce the macrophages to enter the peritoneal cavity. On day 5 post-injection, the rats were anesthetized with sodium pentobarbital (50 mg/kg I.P) and injected with 5 to 10 ml cold PBS with 10 mmol/L EDTA to rinse the macrophages from the peritoneal cavity. The abdominal exude with rinsing liquid was collected, the abdominal exudate was centrifuged at 400 g for 10 minutes. The supernatant was discarded and the pellet was washed by PBS twice, and resuspended in 0.2 ml DMEM, the cells were counted. The macrophages isolated from the peritoneal cavity were plated separately in 6-well tissue culture plates and cultured with RPMI/10% fetal bovine serum (FBS) overnight. The following morning, nonadherent cells were removed by aspiration and washing three times with PBS. .

**Cell culture:**

**HUVECs : Cat #: CRL-1730^TM^,** ATCC, Manassas, VA

# Neutralizing anti-TNFa antibody: Abcam Cat: ab6671

Human umbilical vein endothelial cells (HUVECs, ATCC, Manassas, VA) were cultured in DMEM medium supplemented with 10% FBS. The cells were cultured at 37^o^C, 95% humidity, and 5% CO_2_. All experiments were performed using cells between passages 4 and 12. The cells were seeded in six-well plates (5 x 10^5^ cells/well), and starved in serum free DMEM medium for 24 hours before the experiments were performed. The macrophages (about 10^6^ cells) isolated from the peritoneal cavity were cultured in RPMI/10% FBS for 3 days. The conditioned medium was collected, centrifuged, filtered, and stored at -20 C in aliquots. The remaining macrophages were collected for real-time PCR experiments. The macrophage-conditioned medium was added to HUVECs in final concentration of 20%. For neutralizing experiments, macrophage-conditioned medium was preincubated with neutralizing TNFα antibody (5 μg/ml) for 1 hour. The conditioned medium was then added to HUVECs to incubate for another 24 hours. Following precipitation of the antigen antibody complex, the concentration of TNFα in the media was measured by ELISA to determine efficacy of neutralization.

**Detection of superoxide anion (O_2_^–^) generation in the aorta:**

**Reaction and homogenization buffer:**

**Krebs’ buffer composition in mmol/L:** NaCl 98, KCl 4.7, CaCl_2_ 1.2, MgSO_4_ 1.2 , KH2PO_4_ 1.2, NaHCO_3_ 25, glucose 11.1, HEPES-Na 20, PH 7.4.

**Instrument:** Autolumat LB953 multi-tube luminometer (Berthold Technologies, German)

**Procedures:** The O_2_^–^ generation in fresh aortic rings was determined by chemiluminescence of lucigenin (5 μmol/L) with Krebs’ buffer as previously described. The aorta were cleaned of adherent connective tissue, and cut into aortic rings (3 mm). Krebs’ buffer (composition in mmol/L: NaCl 118, KCl 4.7, CaCl_2_ 1.2, MgSO_4_ 1.2 , KH2PO_4_ 1.2, NaHCO_3_ 25, glucose 11.1, PH 7.4) were freshly prepared. The aortic tissues (about 15 mg) were transferred in the tube with 0.9 ml Krebs’ buffer (reaction buffer), superoxide production was consecutively determined for 20 minutes by autolumat LB953 multi-tube luminometer (Berthold Technologies, German) and trigged by auto-injection of 0.1 ml lucigenin (50 μmol/L, in final concentration of 5 μmol/L, Sigma Aldrich, St Louis, MO). After completion, the aortic tissues were dried in over overnight, the dried aortic tissues were weighted, the result were expressed as counts/min/mg dry tissue. Chemiluminescence of lucigenin has been validated as method to measure O_2_^-^.

# ELISA assay:

# Product information:

# TNFα ELLISA assay kit: Cat#: 941440 (rat TNFα immunoassay)

# CRP ELLISA assay kit: Cat #: DY1744 Rat C-reactive protein ELLISA kit.

# Cotinine assay kit: Cat #: ABIN 2683888 (Cal Biotech)

# Procedures: Plasma levels of C-reactive protein (CRP) and insulin, and TNFα concentration in plasma and conditional media were determined using ELISA assay kits (R&D systems, Minneapolis, Minnesota, USA), following the manufacturer’s instructions, as described in our previous studies. The concentrations were calculated from a standard curve. HOMA-IR, an index for insulin resistance, was calculated by multiple of fasted plasma level of glucose by fasted plasma level of insulin. The plasma level of cotinine, a stable metabolite of nicotine was also determined using Cotinine ELISA kit (Cal Biotech) following the manuscript’s instruction.

### Organ chamber experiments:

### Krebs buffer: composition in mmol/L: NaCl 98, KCl 4.7, CaCl_2_ 1.2, MgSO_4_ 1.2 , KH2PO_4_ 1.2, NaHCO_3_ 25, glucose 11.1, HEPES-Na 20, PH 7.4

**High potassium Krebs buffer:** composition in mmol/L: NaCl 42.7, KCl 60, CaCl_2_ 1.2, MgSO_4_ 1.2 , KH2PO_4_ 1.2, NaHCO_3_ 25, glucose 11.1, HEPES-Na 20, PH 7.4

**Instrument:** 4 channels organ bath system (DMT 720 MO, Denmark)

### Procedures: The aorta was removed and immediately placed into ice-cold modified Krebs-Ringer solution (composition in mmol/L: NaCl 98, KCl 4.7, CaCl_2_ 1.2, MgSO_4_ 1.2 , KH2PO_4_ 1.2, NaHCO_3_ 25, glucose 11.1, HEPES-Na 20, PH 7.4). The vessels were cleaned of adherent connective tissue, and the rings (about 3 mm in length) were cut. A ring was mounted between 2 wires in 5 mL Krebs’ solution of organ bath (37 ^o^C) with consecutive supplement of mixed gas (95% O2 and 5% CO2). The rings were equilibrated under a resting tension of 1 g for at least 90 minutes and were exposed 2 or 3 times to high KCL Krebs’ solution (60 mmol/L KCL, NaCL was replace by equal mmol/L KCL in the solution) at 30 minutes intervals. The rings were pre-contracted by 70% of maximal contraction to norepinephrine for 5 minutes, then an accumulative dose of to acetylcholine (10^-9^ to 10^-5^ mol/L, with 2 minute interval of each dose) was added in organ bath. The concentration of norepinephrin to reach 70% of maximal contraction is based on the preliminary result of average concentration of norepinephrine-induced 70% of maximal constriction force, in most conditions, the concentration of norepinephrine is about 3 x 10 ^-7^ mol/L in rat aortic rings. Contraction or relaxation force of aortic ring were recorded by organ bath system (DMT 720 MO, Denmark). Relaxation of aortic rings was expressed as a percentage inhibition of norepinephrine-induced constriction. Endothelium-dependent relaxation (EDR). The maximal response to acetylcholine (Emax) and the concentration of acetylcholine required for a half-maximal response curve (ED_50_) were determined from the concentration-response curve, using best fit to a logistic sigmoid function.

**Western blot:**

**Antibody information:**

**Goat polyclonal TNFa antibody: Santa Cruz Biotech SC1351**

**Mouse monoclonal b-actin antibody: Santa Cruz Biotech SC47778**

**Rabbit monoclonal eNOS (D9A5L): Cell signaling #32027S**

**Procedures:** Western blotting analysis was used to determine the protein expression of TNFα and eNOS in the aorta. Briefly, the aorta was cut into small pieces with a scissor, then the aortic tissues were placed in a 2 ml tubes with 3 time volumes of homogenization buffer (Lysis buffer) containing 1% protease inhibitor mixture (Sigma-Aldrich), the aortic tissues were homogenized for 45 second with 30 second remission at 4 ^o^C, and the homogenization was repeated three times. After the homogenization, the protein content of the different samples was determined by Bio-Rad protein assay (Life Science, Hercules, CA). Thirty μg of protein was loaded on each lane, separated by SDS-PAGE and transferred to a nitrocellulose membrane. After blocking (TNFα with 5% milk, eNOS with 5% BSA), transferred proteins were incubated overnight with specific primary antibodies against TNFα (1:500 dilution, Cat#: SC1351, Santa Cruz Biotech, Santa Cruz, CA) and eNOS (1:1000 dilution, Cat#: 32027S, Cell Signaling, Danvers, MA). After washing, the blots were incubated with the appropriate secondary antibody and signal detected by luminal chemiluminescence, followed by exposure to an autoradiography film. The membrane was reblotted for β-actin (1:500 dilution,Cat#: SC47778, Santa Cruz Biotech, Santa Cruz, CA) as a loading control.

**Real-time PCR:**

cDNA synthesis kit: I Script cDNA synthesis kit, cat#: 170-889 (Bio-Red)

**Primer information with fluorescence probe: ABI**

CD36 (assay ID: Rn00590726-m1), TNFα (Rn09999017−μ1), interleukin (IL) 1β (Rn01336189-m1),  eNOS (Rn02132634-s1), gp91phox (Rn00576710-m1), p22phox (Rn00577357-m1).

Instrument: Step One Plus real time PCR system, ABI

**PCR running protocol:**

Step 1: 95 ^o^C for 10 minutes

Cycle: 1

Step 2:

95 ^o^C for 15 S

60 ^o^C at 60 S

Cycles: 40

Storage at 4 ^0^C

**Procedures:** HUVECs or the macrophages from the peritoneal cavity were harvested in 1 ml trizol reagent, the aortic tissues were homogenated in 1 ml trizol reagent. Total RNA (2 µg) for each sample were reverse-transcribed into cDNA using a superscript II RT first strand synthesis kit (Gibco, BRL) in final volume of 24 μl according to the manufacturer’s instructions. One microliter of cDNA (about 80 ng) was ampilified in 18 μl of TaqMan master assay buffer (ABI) with 1 μl of PCR primer with fluorescence probe. Sample were heated for 10 minutes at 94 ^o^C, then subjected to 15 s at 95 ^o^C and 1 minute at 60 ^o^C for 40 cycles (Step One Plus real time PCR system, ABI). Relative quantities of each transcript were normalized by a housekeeping gene (GAPDH) and expressed as fold increase vs. control. All PCR primers with fluorescence probe for CD36 (assay ID: Rn00590726-m1), TNFα (Rn09999017−μ1), interleukin (IL) 1β (Rn01336189-m1),  eNOS (Rn02132634-s1), gp91phox (Rn00576710-m1), p22phox (Rn00577357-m1) were ordered through ABI online system with assay IDs.

**PCR array for determination of mRNA profile of rat inflammatory signaling pathway:**

**PCR array kit:** Rat inflammatory cytokine & receptor signaling pathway, Cat #: PARN-011Z (SupperArray, Qiagen)

The aortic tissues were homogenated in 1 ml trizol reagent, one μg of RNA was converted to cDNA with random primers in a 20-μl reaction volumes using high capacity cDNA archive kit (C-3, Superarray). The cDNA was diluted in total volume 100 μl. One μl of cDNA was used for each primer set in the PCR array according to the manufacturer’s instruction as previously described. Rat inflammatory cytokine & receptor signaling pathway was used to determine a panel of inflammatory gene expression (PARN-011Z, SuperArray Qiagen). This PCR array includes 84 target genes of inflammatory chemokines, cytokines, interleukins and their receptors and 12 control genes. The control genes include each array for genomic DNA contamination, RNA quality, housekeep and general PCR performance. Data analysis was performed using the manufacturer’s integrated web-based software package for the PCR array system using delta-delta Ct based fold-change calculations and normalized by a housekeeping control gene.
